# Effects of homocysteine lowering with B vitamins on cognitive aging: meta-analysis of 11 trials with cognitive data on 22,000 individuals^1^,^2^,^3^,^4^,^5^

**DOI:** 10.3945/ajcn.113.076349

**Published:** 2014-06-25

**Authors:** Robert Clarke, Derrick Bennett, Sarah Parish, Sarah Lewington, Murray Skeaff, Simone JPM Eussen, Catharina Lewerin, David J Stott, Jane Armitage, Graeme J Hankey, Eva Lonn, J David Spence, Pilar Galan, Lisette C de Groot, Jim Halsey, Alan D Dangour, Rory Collins, Francine Grodstein, on behalf of the B-Vitamin Treatment Trialists’ Collaboration

**Affiliations:** 1 From the Clinical Trial Service Unit and Epidemiological Studies Unit, University of Oxford, Oxford, United Kingdom (R Clarke, DB, SP, SL, JA, JH, and R Collins); the Department of Human Nutrition, University of Otago, Dunedin, New Zealand (MS); the Section for Pharmacology and Department of Public Health and Primary Care, University of Bergen, Bergen, Norway (SJPME); the Department of Epidemiology, School for Public Health and Primary Care, CAPHRI, Maastricht University Medical Centre, Maastricht, Netherlands (SJPME); the Section of Hematology and Coagulation, Department of Internal Medicine, Institute of Medicine, Sahlgrenska Academy at the University of Gothenburg, Gothenburg, Sweden (CL); the Division of Cardiovascular and Medical Science, University of Glasgow, Glasgow, United Kingdom (DJS); the School of Medicine and Pharmacology, The University of Western Australia, Perth, Australia (GJH); the Population Health Research Institute and Department of Medicine, McMaster University, Hamilton, Canada (EL); the Department of Neurology, Western University, London, Canada (JDS); Unité de Recherche en Epidémiologie Nutritonnelle (UREN), Sorbonne-Paris-Cité, UMR Inserm U557, France (PG); Inra U1125, Paris, France (PG); Cnam, Paris, France (PG); Université Paris 13, CRNH IdF, Bobigny, France (PG); the Division of Human Nutrition and Epidemiology, Wageningen University, Wageningen, Netherlands (LCdG); the Department of Nutrition and Public Health Intervention Research, London School of Hygiene and Tropical Medicine, London, United Kingdom (ADD); and the Channing Division of Network Medicine, Department of Medicine, Brigham and Women's Hospital, Boston, MA (FG).

## Abstract

**Background:** Elevated plasma homocysteine is a risk factor for Alzheimer disease, but the relevance of homocysteine lowering to slow the rate of cognitive aging is uncertain.

**Objective:** The aim was to assess the effects of treatment with B vitamins compared with placebo, when administered for several years, on composite domains of cognitive function, global cognitive function, and cognitive aging.

**Design:** A meta-analysis was conducted by using data combined from 11 large trials in 22,000 participants. Domain-based *z* scores (for memory, speed, and executive function and a domain-composite score for global cognitive function) were available before and after treatment (mean duration: 2.3 y) in the 4 cognitive-domain trials (1340 individuals); Mini-Mental State Examination (MMSE)–type tests were available at the end of treatment (mean duration: 5 y) in the 7 global cognition trials (20,431 individuals).

**Results**: The domain-composite and MMSE-type global cognitive function *z* scores both decreased with age (mean ± SE: −0.054 ± 0.004 and −0.036 ± 0.001/y, respectively). Allocation to B vitamins lowered homocysteine concentrations by 28% in the cognitive-domain trials but had no significant effects on the *z* score differences from baseline for individual domains or for global cognitive function (*z* score difference: 0.00; 95% CI: −0.05, 0.06). Likewise, allocation to B vitamins lowered homocysteine by 26% in the global cognition trials but also had no significant effect on end-treatment MMSE-type global cognitive function (*z* score difference: −0.01; 95% CI: −0.03, 0.02). Overall, the effect of a 25% reduction in homocysteine equated to 0.02 y (95% CI: −0.10, 0.13 y) of cognitive aging per year and excluded reductions of >1 mo per year of treatment.

**Conclusion:** Homocysteine lowering by using B vitamins had no significant effect on individual cognitive domains or global cognitive function or on cognitive aging.

## INTRODUCTION

Cognitive function, and its component domains of memory, speed, and executive function, decline gradually over the life span in most people (1). The rate of decline in cognitive function with increasing age is faster in some people, resulting in clinical syndromes of “mild cognitive impairment” and dementia (including Alzheimer disease) (1). With improvements in life expectancy, the number of cases with mild cognitive impairment and dementia is likely to increase worldwide. Observational studies have shown that elevated plasma homocysteine is a potentially modifiable risk factor for “cognitive aging” (2–6). The “homocysteine hypothesis” of Alzheimer disease was suggested in response to observations from retrospective studies that cases with clinically diagnosed or histologically confirmed Alzheimer disease had higher homocysteine concentrations compared with age- and sex-matched controls (2, 3). Subsequently, prospective studies in healthy older people reported that individuals with homocysteine concentrations ≥14 μmol/L had a 2-fold higher risk of Alzheimer disease after adjustment for known risk factors (5, 6). The results of these studies (2, 4–6) prompted the design of several randomized trials, testing whether dietary supplementation with folic acid and vitamin B-12 to lower homocysteine concentrations could slow the rate of age-related cognitive decline and thereby reduce the risk of dementia, including Alzheimer disease.

A few trials, typically involving several hundred people, assessed the effects of B vitamins administered for a few years on domain-specific tests of cognitive function (ie, memory, speed, and executive function and their sum, domain-composite score) before and after treatment (7–10). Other trials, typically involving several thousand individuals, assessed the effects of B vitamins administered for ∼5 y on cardiovascular disease outcomes (11–17) and included some assessments of global cognitive function [typically assessed by using the Mini-Mental State Examination (MMSE)^6^ (18) or the Telephone Interview for Cognitive Status–Modified (TICS-M) (19–21)] at the end of the treatment period.

The B-Vitamin Treatment Trialists’ Collaboration was established to conduct meta-analyses of data from individual participants in placebo-controlled trials assessing the effects of supplementation with B vitamins on cardiovascular disease, cancer, and cognitive function (22). The primary aims of the present meta-analysis were to evaluate the effects of homocysteine lowering by B-vitamin treatment on cognitive function and on the rate of cognitive aging. Treatment effects were expressed both as B vitamins compared with placebo differences and as differences by extent of homocysteine lowering. When trial data permitted, effects on changes in specific cognitive domains were assessed and effect modification by a variety of factors (eg, age, sex, duration of treatment, smoking, prior stroke or cognitive impairment, folic acid fortification, and baseline plasma concentrations of folate, vitamin B-12, and homocysteine) was also evaluated.

## METHODS

### Trial eligibility

Randomized trials were sought by 2 investigators (R Clarke and DB) who searched electronic databases, including PubMed (www.ncbi.nlm.nih.gov/pubmed) and PsychINFO (www.ebscohost.com/academic/psycinfo), with the use of search terms “cognitive function,” “cognitive impairment,” “cognitive decline,” “memory” and “memory impairment,” and “folic acid” or “B-vitamins” or “homocysteine lowering therapy” for reports in the English language (Supplemental Figure 1 under “Supplemental data” in the online issue). Unpublished trials were sought through electronic searches, hand-searching reference lists of relevant reports and discussions with experts in the field. Nine of ten randomized trials assessing the effects on cognitive function of supplementation with B vitamins containing folic acid met the following criteria: *1*) duration of >3 mo, *2*) >100 participants unselected for cognition-related diseases other than heart attack or stroke/transient ischemic attack (TIA), *3*) homocysteine-lowering treatment only, and *4*) availability of sufficient data by September 2010. The Folic Acid and Carotid Intima-Media Thickness (FACIT) trial declined to contribute individual participant data, but the published results were sufficiently detailed to allow the trial's inclusion (10).

### Baseline and follow-up data

For each randomly assigned participant, information was sought on characteristics recorded before randomization, the randomly allocated treatment, the dates of randomization and follow-up visits (or time from randomization), and results of any measure of cognitive function before, during, and at the end of the scheduled treatment period. The individual participant data were checked for consistency with any published reports, and investigators were also asked to confirm summary data by allocated treatment to help ensure that the data were incorporated correctly into the meta-analysis.

### Measures of cognitive function

Four trials (7–10) assessed effects of B-vitamin treatment on specific cognitive domains, and 7 assessed effects of B-vitamin treatment on MMSE-type global cognitive function scores (**Table 1** and Supplemental Table 1 under “Supplemental data” in the online issue). Each of the 4 “cognitive-domain trials” used multiple domain-specific tests that were combined by a standard approach to yield composite scores for memory, speed, executive function, and global cognitive function (Table 1 and Supplemental Table 1 and Supplemental Methods under “Supplemental data” in the online issue). Each of the 7 “global cognition trials” (11–17) typically used a single test of global cognitive function: the MMSE, the Telephone Interview for Cognitive Status (TICS), or the TICS-M.

**TABLE 1:** Design and eligibility criteria of included trials*^1^*

									Difference by allocated treatment			
			Subjects with cognitive function measured		Domain tests		MMSE-type test		Folate	tHcy		
Trial (ref)	Age*^2^*	Duration of treatment	B	E	Memory/speed/executive function*^3^*	Available at B and E	Type	Available at B and E	Difference*^4^*	No. with tHcy	tHcy reduction*^5^*	Equivalent study years at 25% tHcy reduction*^6^*
	*y*	*y*	*n*	*n (%)*	*No. of tests (total)*				*nmol/L*		*%*	*y*
Cognitive-domain trials												
Eussen (7)*^7^*	82 ± 5	0.5	130	108 (83)	6/ 5/ 7 (18)	Y	MMSE	Y	—	105	34.9 (3.5)	0.64 (0.06)
Lewerin (8)	76 ± 5	0.3	202	183 (91)	4/ 4/ 2 (10)	Y	—	—	47.0 (16)	193	32.1 (2.7)	0.39 (0.03)
McMahon (9)	74 ± 6	2.2	273	248 (91)	3/ 1/ 3 (7)	Y	MMSE	Y	58.1 (41)	253	31.9 (2.2)	2.84 (0.20)
FACIT (10)*^8^*	60 ± 6	3.0	818	801 (98)	3/ 7/ 1 (11)	Y	MMSE	Y	62.9 (53)	798	26.1 (1.2)	3.13 (0.15)
Subtotal	68 ± 5	2.3	1423	1340 (94)	—	—	—	—	53.3 (41)	1349	28.4 (0.7)	—
Global cognition trials												
Stott (11)*^7^*	74 ± 6	1.0	185	167 (90)	0/ 1/ 0 (1)	—	TICS-M	Y	—	175	29.8 (3.4)	1.13 (0.13)
HOPE-2 (12)	69 ± 7	4.8	1327	1245 (94)	0/ 0/ 0 (0)	—	MMSE	Y	23.5 (32)	580	24.1 (1.8)	4.64 (0.35)
SU.FOL.OM3 (13)*^7^*	61 ± 9	4.5	—	1309 (—)	0/ 0/ 0 (0)	—	F-TICS-M	N	21.9 (29)	835	21.7 (1.6)	3.88 (0.29)
WAFACS (14)	71 ± 4	6.0	2007	1756 (87)	4/ 0/ 1 (5)	—	TICS	Y	55.2 (51)	106	12.4 (4.4)	2.95 (1.04)
VISP (15)	66 ± 11	1.8	3680	2653 (72)	0/ 0/ 0 (0)	—	MMSE	Y	40.7 (58)	3006	18.0 (0.8)	1.30 (0.06)
VITATOPS (16)	63 ± 13	3.2	—	4410 (—)	0/ 0/ 0 (0)	—	MMSE	N	9.7 (18)	925	25.7 (1.5)	3.32 (0.19)
SEARCH (17)	63 ± 9	7.1	—	8891 (—)	0/ 0/ 1 (1)	—	TICS-M	N	38.7 (36)	8289	29.6 (0.5)	8.45 (0.14)
Subtotal	66 ± 6	5.0	7199	20,431 (284)	—	—	—	—	29.0 (46)	13,916	26.1 (0.3)	—

1 B, baseline; E, end of treatment; FACIT, Folic Acid and Carotid Intima-Media Thickness; F-TICS-M, French Telephone Interview for Cognitive Status-Modified; HOPE-2, Heart Outcomes Prevention Evaluation-2; MMSE, Mini-Mental State Examination; N, no; ref, reference; SEARCH, Study of the Effectiveness of Additional Reductions in Cholesterol and Homocysteine; SU.FOL.OM3, Supplementation with Folate, vitamin B6 and B12 and/or Omega-3 fatty acids; tHcy, total homocysteine; TICS, Telephone Interview for Cognitive Status; TICS-M, Telephone Interview for Cognitive Status–Modified; VISP, Vitamin Intervention for Stroke Prevention; VITATOPS, Vitamins to Prevent Stroke; WAFACS, Women's Antioxidant and Folic Acid Cardiovascular Study; Y, yes.

2 Values are means ± SDs.

3 For the study by Eussen, speed and praxis tests were combined.

4 Values are medians (IQRs) and are based on individuals who had measurements at both baseline and follow-up.

5 Values are percentages (SEs) and are based on individuals who had measurements at both baseline and follow-up.

6 Values are means (SEs). Equivalent study years at 25% tHcy reduction = (% tHcy reduction/25) × duration of treatment.

7 Refers to group-allocated B vitamins compared with placebo.

8 Published data only.

The MMSE is a brief test of cognitive function involving 5 sections (orientation, immediate and delayed recall, attention and calculation, language, and visuospatial abilities). The TICS and TICS-M are telephone adaptations of the MMSE. The 13-item TICS-M includes 4 sections (orientation; recent and delayed recall; attention and calculation; semantic memory, comprehension, and language). The MMSE-type cognitive function tests typically provide a single score, but for the Study of the Effectiveness of Additional Reductions in Cholesterol and Homocysteine (SEARCH) trial (17) the 4 components of the TICS-M test were also available, permitting additional assessments of individual domains. (Note that terminology, and in particular the use of the term “global,” has varied in previous reports, but because both types of test cover multiple domains the overall score from both types of test is termed a “global cognitive score” in this article.)

### Statistical analyses

Homocysteine reductions were estimated from regression of follow-up blood homocysteine concentrations (in the blood samples taken nearest the midpoint of the trial when multiple replicate samples were available) on allocated treatment after adjustment for baseline homocysteine concentration. To test the hypothesis that homocysteine lowering attenuates the annual rate of cognitive aging pro rata to the percentage of homocysteine reduction and duration of reduction, the equivalent study years at a 25% homocysteine reduction for each trial were estimated by dividing the trial-specific percentage of homocysteine reduction by 25 and multiplying by the mean duration of treatment in that trial (23).

Because the cognitive tests and populations studied differed, scores from each trial were rescaled, as follows: first the residual SDs of the end-treatment domain-specific scores, the domain-composite global cognitive function scores, and the MMSE-type global cognitive function scores were estimated after adjustment for end-treatment age (as a continuous variable by using linear regression analysis); then, the before- and after-treatment scores were each scaled by dividing by the estimated residual SD (Supplemental Table 2 under “Supplemental data” in the online issue).

Standard linear models and Pearson correlation coefficients were used to compute all statistics on the *z* scores. Self-correlations were calculated as the Pearson correlation in participants with global cognitive *z* scores measured at baseline and at end of treatment. Preliminary analyses compared the properties of domain-composite and MMSE-type global cognitive function scores and the effects of age and prior stroke or TIA on the *z* scores.

For the main comparisons, end-treatment MMSE-type global cognitive function scores were adjusted for age to remove some between-person variation, whereas this was not relevant to comparisons of changes in *z* scores in the trials. All comparisons were conducted separately within each trial and the trial-specific estimates subsequently combined by using inverse-variance–weighted averaging.

The *z* score differences per year at a 25% homocysteine reduction were estimated by dividing the study *z* score difference by the trial equivalent years at a 25% homocysteine reduction. These estimates were then divided by the effect of age on the respective global cognitive function score (domain-composite or MMSE-type) estimated over all trials with that score (to provide equivalent years of cognitive aging). Additional details of how the summary results from the FACIT trial were incorporated are provided in the Supplementary Material: Methods Appendix (under “Supplemental data” in the online issue). All analyses used SAS version 9.2 (SAS Institute).

## RESULTS

### Characteristics of the participating trials

The 4 cognitive-domain trials reported results for domain-specific and domain-composite global cognitive function scores in 1423 individuals, of whom 1340 (94%) had complete cognitive data at the end of the scheduled treatment period (Table 1 and Supplemental Table 1 under “Supplemental data” in the online issue). The 7 global cognition trials reported MMSE-type scores in 20,431 participants at end of treatment (with 7199 of these individuals also having MMSE-type scores recorded before starting treatment). The mean duration of treatment varied from 0.3 to 3.0 y (overall mean: 2.3 y) in the 4 cognitive-domain trials and from 1.0 to 7.1 y (overall mean: 5.0 y) in the global cognition trials. All trials compared the effects of folic acid with placebo, except for one trial (13) that used 5-methyltetrahydrofolate. The daily doses of folic acid ranged from 0.4 to 2.5 mg (Supplemental Table 3 under “Supplemental data” in the online issue). All but one trial (10) also added vitamin B-12 (dose range: 0.02–1 mg). The mean (±SD) age at entry was 68 ± 5 y in the cognitive-domain trials and 66 ± 6 y in the global cognition trials (Table 1).

### Effects on plasma homocysteine concentrations

Allocation to B vitamins was associated with a 28.4% and 26.1% reduction in plasma concentrations of homocysteine in the cognitive-domain trials and global cognition trials, respectively (Table 1). Allocation to B vitamins was associated with an approximate 3- to 5-fold increase in median plasma folate concentrations (Supplemental Table 4 under “Supplemental data” in the online issue) and a 2-fold increase in median vitamin B-12 concentrations in all trials, which showed a high level of compliance with the allocated treatment (Supplemental Table 5 under “Supplemental data” in the online issue).

### Characteristics of the instruments used to assess cognitive function

The correlations and variances of global cognitive function scores in placebo-allocated participants with before- and after-treatment (ie, repeat) assessments, with the trials ordered by duration of treatment within the trial categories, are shown in **Table 2**. The correlation between baseline and end-treatment scores was very high (0.92–0.86) for the domain-composite score, but trials with this information had a limited duration of treatment (range: 0.3–2.2 y). Correspondingly, the change in score over a trial had a substantially lower variance than that of a single-score measurement adjusted for age, with the ratios of variances ranging from 0.16 to 0.38 in studies with treatment durations of up to 3 y [including published results from FACIT (10)].

**TABLE 2:** Correlations and variance of global cognitive function scores in placebo-allocated participants with repeat assessments*^1^*

		Global cognitive function score								
		Domain-composite				MMSE-type				
Trial (ref)	Duration	Self-correlation	V_E_	V_C_	V_C_:V_E_ ratio	Self-correlation	V_E_	V_C_	V_C_:V_E_ ratio	Correlation between domain-composite and MMSE-type score
	*y*									
Cognitive-domain trials										
Lewerin (8)	0.3	0.92	1.00	0.16	0.16					
Eussen (7)	0.5	0.90	1.00	0.43	0.43	0.75	1.00	0.48	0.48	0.63
McMahon (9)	2.2	0.86	1.00	0.27	0.27	0.40	1.00	1.29	1.29	0.49
FACIT (10)*^2^*	3.0		1.00	0.23	0.23					
Global cognition trials										
Stott (11)	1.0					0.76	1.00	0.50	0.50	
VISP (15)	1.8					0.54	1.00	0.87	0.87	
HOPE-2 (12)	4.8					0.53	1.00	0.92	0.92	
WAFACS (14)*^3^*	6.0					0.41	1.00	1.13	1.13	

1 FACIT, Folic Acid and Carotid Intima-Media Thickness; HOPE-2, Heart Outcomes Prevention Evaluation-2; MMSE, Mini-Mental State Examination; ref, reference; V_C_, variance of change in *z* score from baseline to end of treatment; V_E_, variance of end-treatment *z* score (adjusted for age); VISP, Vitamin Intervention for Stroke Prevention; WAFACS, Women's Antioxidant and Folic Acid Cardiovascular Study.

2 Published data only.

3 WAFACS also measured cognitive function at 2 and 4 y after the initial measurement, and the self-correlations over these intervals were 0.47 and 0.43.

Two of the cognitive-domain trials also measured an MMSE-type cognitive test; and in each trial, the self-correlation of the MMSE-type scores (0.40 and 0.75) was lower than the self-correlation of the domain-composite score. In the trials with durations of ∼2 y or more, the variance of the change in the MMSE-type score was very similar to the variance of a single age-adjusted score at the end of the trial. Domain-composite and MMSE-type global cognitive function scores were moderately correlated with each other in the 2 trials that assessed this (0.49 and 0.63).

### Effects of age on cognitive function

The relation of the global cognitive function scores with age and history of stroke or TIA is shown in **Table 3**. Among individuals selected for entry into these trials, the proportion of variance explained by age in the different trials was fairly low (varying from 2% to 13% for the MMSE-type test). In trials that did not primarily recruit participants with prior stroke/TIA, both the domain-composite and MMSE-type global cognitive function scores declined significantly with age, but the relation was 50% stronger with the domain-composite score (mean ± SE: −0.054 ± 0.004 compared with −0.036 ± 0.001/y). The effects in the individual trials were consistent with the overall estimates, except for a weaker effect seen in the Heart Outcomes Prevention Evaluation-2 trial (12) (Table 3). After adjustment for age, history of stroke/TIA was associated with a −0.135 ± 0.027 lower score (ie, equivalent to ∼4 y of aging) in all trials in participants with a prior history of stroke or TIA, but the proportion of variance explained by history of stroke/TIA was also low (0–3%) due to the low prevalence of stroke at baseline.

**TABLE 3:** Effects of age (per year) and prior history of stroke/TIA on the domain-composite and MMSE-type cognitive scores*^1^*

						Domain-composite		MMSE-type			
						Age		Age		Stroke/TIA*^3^*	
Trial (ref)	Subjects with cognitive function measured at baseline	Subjects with cognitive function at end of treatment	Duration of treatment	Age at baseline*^2^*	No. of strokes/TIAs	Effect (SE)	Variance	Effect (SE)	Variance	Effect (SE)	Variance
	*n*	*n (%)*	*y*	*y*			*%*		*%*		*%*
Cognitive-domain trials											
Eussen (7)	130	108 (83)	0.5	82 ± 5	7	−0.055 (0.020)	14	−0.063 (0.016)	13	0.292 (0.373)	0.0
Lewerin (8)	202	183 (91)	0.3	76 ± 5	—	−0.055 (0.016)	6	—	—	—	—
McMahon (9)	273	248 (91)	2.2	74 ± 6	—	−0.075 (0.010)	19	−0.035 (0.010)	4	—	—
FACIT (10)*^4^*	818	801 (98)	3.0	60 ± 6	—	−0.048 (0.005)	—	—	—	—	—
Global cognition trials											
Global cognition trials primarily in participants without a prior history of stroke or TIA											
HOPE-2 (12)	1327	1245 (94)	4.8	69 ± 7	140			−0.020 (0.004)	2	0.101 (0.090)	0.1
SU.FOL.OM3 (13)	—	1309 (—)	4.5	61 ± 9	490			−0.036 (0.003)	9	−0.301 (0.054)	2.6
WAFACS (14)	2007	1756 (87)	6.0	71 ± 4	505			−0.038 (0.005)	3	−0.040 (0.053)	0.1
SEARCH (17)	—	8891 (—)	7.1	63 ± 9	523			−0.037 (0.001)	10	−0.151 (0.043)	0.3
All trials above						−0.054 (0.004)		−0.036 (0.001)		−0.135 (0.027)	
Heterogeneity over all above*^5^*						χ^2^_3_ = 5.9, *P* = 0.11		χ^2^_5_ = 21.2, *P* = 0.002^6^		χ^2^_4_ = 21.0, *P* = 0.0003	
Global cognition trials primarily in participants with a prior history of stroke or TIA											
Stott (11)*^7^*	185	167 (90)	1.0	74 ± 6	—			−0.021 (0.011)	2	—	—
VISP (15)*^7^*	3680	2653 (72)	1.8	66 ± 11	3681			−0.012 (0.002)	2	—	—
VITATOPS (16)*^7^*	—	4410 (—)	3.2	63 ± 13	8164			−0.015 (0.001)	3	—	—
All stroke/TIA trials								−0.014 (0.001)			
Heterogeneity over all stroke/TIA trials*^5^*								χ^2^_2_ = 2.4, *P* = 0.30			

1 FACIT, Folic Acid and Carotid Intima-Media Thickness; HOPE-2, Heart Outcomes Prevention Evaluation-2; MMSE, Mini-Mental State Examination; ref, reference; SEARCH, Study of the Effectiveness of Additional Reductions in Cholesterol and Homocysteine; SU.FOL.OM3, Supplementation with Folate, vitamin B6 and B12 and/or Omega-3 fatty acids; TIA, transient ischemic attack; VISP, Vitamin Intervention for Stroke Prevention; VITATOPS, Vitamins to Prevent Stroke; WAFACS, Women's Antioxidant and Folic Acid Cardiovascular Study.

2 Values are means ± SDs.

3 Effect of stroke/TIA adjusted for age

4 Based on published data.

5 χ*_n_*^2^ = chi-square statistic with number of df (*n*) as a subscript.

6 When excluding HOPE-2 in addition to the 3 studies with a prior history of stroke/TIA, the effect per year of age (SE) was −0.037 (0.001) with heterogeneity χ_4_^2^ = 3.2, *P* = 0.67.

7 Approximately 60% of participants in the trial by Stott reported a prior history of stroke (but individual participant data on stroke history were not provided). All of the participants in the VISP trial had a prior history of stroke. All of the participants in the VITATOPS had prior stroke or TIA.

### Effects of B vitamins on domain-specific scores

**FIGURE 1.**
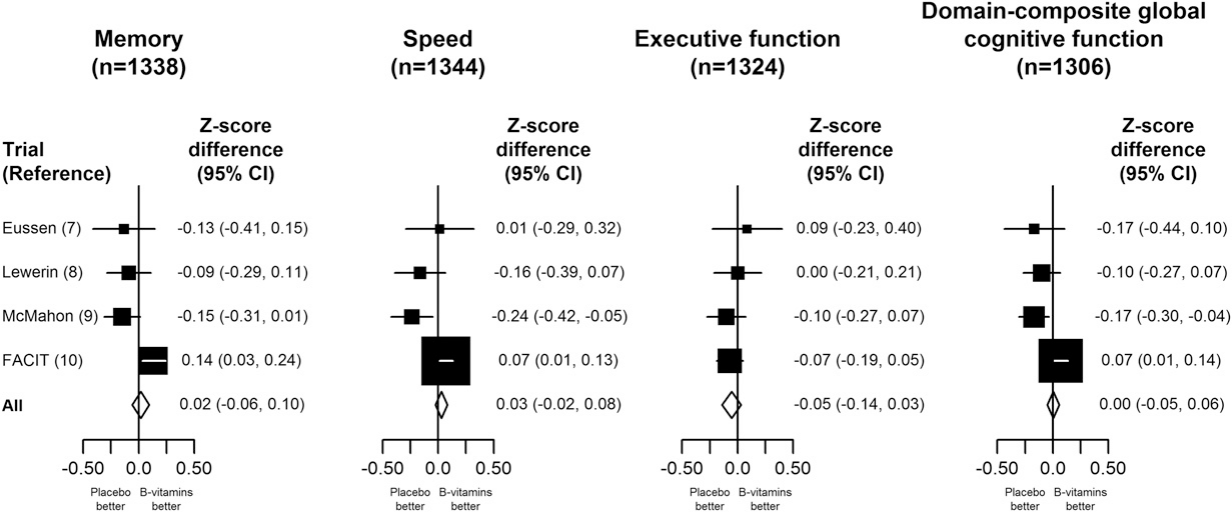
Effects of B vitamins on specific domains of cognitive function and on a domain-composite score by using change from baseline in the cognitive-domain trials. The *z* scores for individual trials and the total for the cognitive-domain trials are shown separately for memory, speed, and executive function and for a domain-composite global cognitive function score. Black squares represent *z* score differences for the individual trials and the horizontal lines represent 95% CIs. The size of the squares is inversely proportional to the variance. Diamonds represent the *z* scores and 95% CIs for all trials.

The effect of B vitamins on specific domains of cognitive function and on the domain-composite global cognitive function score using change from baseline in the cognitive-domain trials is shown in **Figure 1**. Overall, allocation to B vitamins had no significant effect on the changes in the domain-specific scores for memory [*z* score difference: 0.02 (95% CI: −0.06, 0.10); speed: 0.03 (95% CI: −0.02, 0.08); executive function: −0.05 (95% CI: −0.14, 0.03)] or the domain-composite score (0.00; 95% CI:−0.05, 0.06). Neither were there any significant effects of B vitamins on the memory or executive function domains of cognitive function when the data from the cognitive-domain trials were combined with additional data from the Women's Antioxidant and Folic Acid Cardiovascular Study (14) and SEARCH (17) trials that had such data (Supplemental Figure 2 under “Supplemental data” in the online issue). Among the cognitive-domain trials, there was significant heterogeneity between the trials for the effects of B vitamins on memory (χ^2^_3_ = 11.3, *P* = 0.01), speed (χ^2^_3_ = 12.3, *P* = 0.006), and domain-composite global cognitive function (χ^2^_3_ = 13.6, *P* = 0.004) but not for executive function (χ^2^_3_ = 1.4, *P* = 0.71), with the heterogeneity being chiefly attributable to the FACIT trial.

### Effects of B vitamins on MMSE-type scores

**FIGURE 2.**
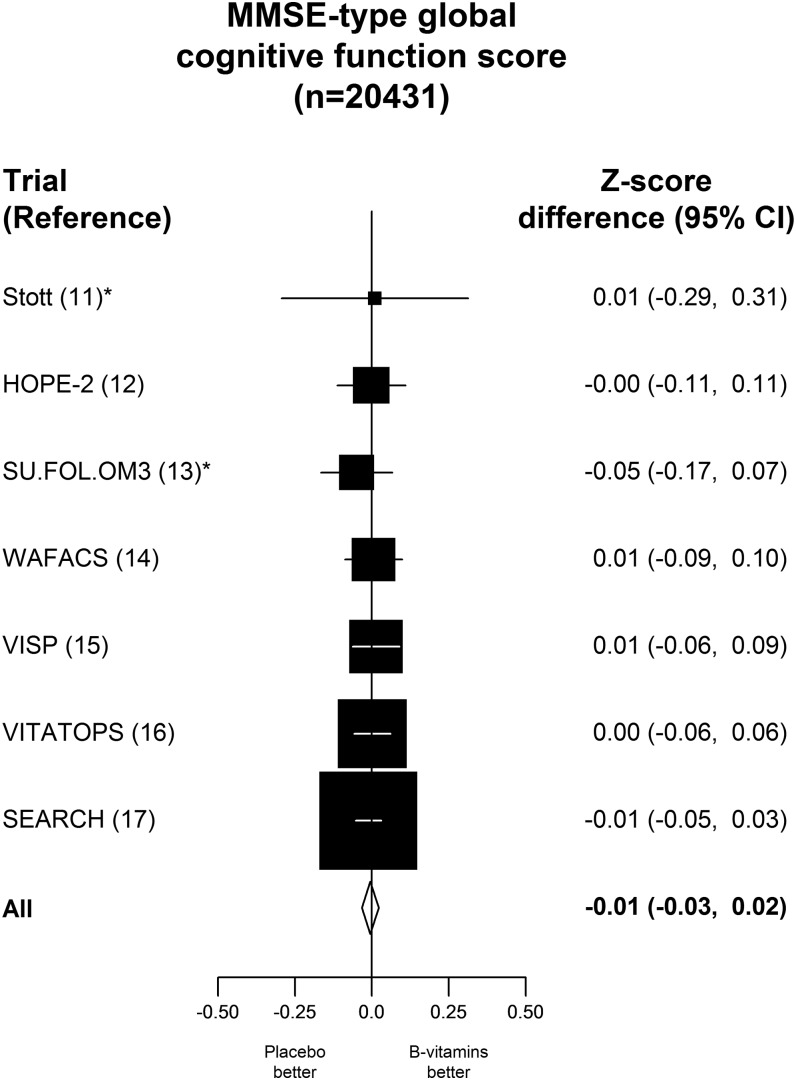
Effects of B vitamins on MMSE-type global cognitive function score at the end of the treatment period in the global cognition trials. The *z* score for differences and their 95% CIs for MMSE-type global cognitive function scores are shown for individual trials and as the total for all trials. The *z* score differences are for B-vitamins compared with placebo. * refers to B-vitamin based treatment vs placebo. HOPE-2, Heart Outcomes Prevention Evaluation-2; MMSE, Mini-Mental State Examination; SEARCH, Study of the Effectiveness of Additional Reductions in Cholesterol and Homocysteine; SU.FOL.OM3, Supplementation with Folate, vitamin B6 and B12 and/or Omega-3 fatty acids; VISP, Vitamin Intervention for Stroke Prevention; VITATOPS, Vitamins to Prevent Stroke; WAFACS, Women's Antioxidant and Folic Acid Cardiovascular Study.

The effect of B vitamins on MMSE-type global cognitive function scores at the end of the treatment period in the global cognition trials is shown in **Figure 2**. Compared with the placebo control, allocation to B vitamins had no significant effect on MMSE-type score in the 20,431 individuals with data on cognitive function (*z* score difference: −0.01; 95% CI: −0.03, 0.02), nor was there any effect of B vitamins on MMSE-type global cognitive function in the cognitive-domain trials (Supplemental Figure 3 under “Supplemental data” in the online issue). Moreover, there was no significant effect of B vitamins on MMSE-type score in any of the subgroups considered, including age at randomization (albeit only one-third were aged >70 y), sex, smoking status, history of stroke, folic acid fortification, duration of treatment, and by approximate thirds of pretreatment concentrations of folate, vitamin B-12, and homocysteine or by presence or absence of cognitive impairment at baseline (Supplemental Figure 4 under “Supplemental data” in the online issue). (Individuals were defined as having cognitive impairment at baseline if they had an MMSE score <24 or a TICS score <31 or a TICS-M score <22). In particular, there was no heterogeneity in the effect of treatment on score even among individuals with folate concentrations <10 nmol/L, with vitamin B-12 concentrations <250 pmol/L, or with homocysteine concentrations ≥15 μmol/L compared with those with normal plasma concentrations of these markers of B-vitamin status (Supplemental Figure 4 under “Supplemental data” in the online issue).

**FIGURE 3.**
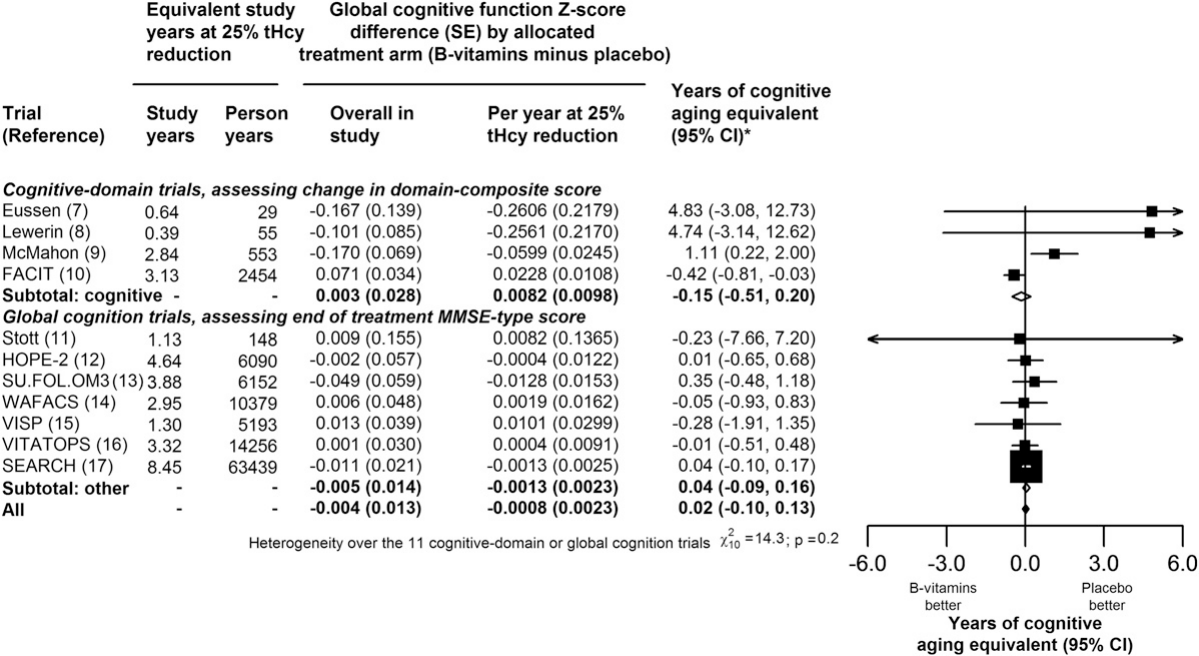
Effects of B vitamins on cognitive aging in all available trials per year at a 25% reduction in homocysteine. The *z* score differences and their 95% CIs are provided for the domain-composite global cognitive function score in each cognitive-domain trial and for the MMSE-type global cognitive function score in the other trials. The years of cognitive aging equivalent and their 95% CIs are also provided for individual trials and their totals. The years of cognitive aging equivalent were determined on the basis of a 0.054/y reduction in the cognitive domain trial score and a 0.036/y reduction in the global cognition trial score. * indicates that the age association was based on 0.054 per year reduction in domain-composite *z* score for cognitive-domain trials and on 0.036 per year reduction in MMSE-type cognitive *z* score. FACIT, Folic Acid and Carotid Intima-Media Thickness; HOPE-2, Heart Outcomes Prevention Evaluation-2; MMSE, Mini-Mental State Examination; SEARCH, Study of the Effectiveness of Additional Reductions in Cholesterol and Homocysteine; SU.FOL.OM3, Supplementation with Folate, vitamin B6 and B12 and/or Omega-3 fatty acids; tHcy, total homocysteine; VISP, Vitamin Intervention for Stroke Prevention; VITATOPS, Vitamins to Prevent Stroke; WAFACS, Women's Antioxidant and Folic Acid Cardiovascular Study.

Information on the effect of B vitamins on global cognitive function from the 4 trials with the use of change in the domain-composite score and the 7 trials that used end-treatment MMSE-type scores is shown in **Figure 3**. The score differences by allocated treatment with B vitamins were also expressed as differences per year at a 25% homocysteine reduction and, by using the score type–specific relations with age provided in Table 3, as years of cognitive aging equivalent. The overall estimates of the effect of B vitamins on global cognitive function were not significant when combined by any of these approaches (Figure 3 and Supplemental Figure 5 under “Supplemental data” in the online issue)**.** The weighted average effect per year of a 25% homocysteine reduction with B vitamins was 0.02 y (95% CI: −0.10, 0.13 y) of cognitive aging (Figure 3) and there was no significant heterogeneity in this effect between studies (χ^2^_10_ = 14.3, *P* = 0.2).

## DISCUSSION

The present meta-analysis in 22,000 participants from 11 randomized trials has shown that homocysteine lowering by dietary supplementation with B vitamins for ∼5 y does not have an impact on cognitive aging in older people with or without vascular disease. Allocation to B vitamins had no material effect on domain-specific and domain-composite scores in the 4 cognitive trials or on end-treatment MMSE-type cognitive scores in 7 global cognition trials, and there was no evidence of effect modification by pretreatment concentrations of folate, vitamin B-12, and homocysteine. With access to individual results on 20,000 participants (together with the availability of sufficient detail in the published report from FACIT), the present report was based on an additional 60,000 equivalent person-years at a 25% homocysteine reduction than the previous meta-analysis (10) and permitted a combined analysis of data from all available trials that used cognitive aging. Overall, in all trials combined, the weighted average effect of a 25% homocysteine reduction equated to 0.02 y (95% CI: −0.10, 0.13 y) of cognitive aging per year, with the lower CI excluding an effect greater than ∼1 mo less aging.

In contrast with previous meta-analyses that addressed the effects of B vitamins on cognitive function or dementia (24, 25), the present meta-analysis took account of differences in the duration of treatment and extent of homocysteine reduction when assessing the effects of B vitamins on cognitive aging. The approach adopted for the analyses of cognitive aging used all of the available randomized evidence for the effects of B vitamins on cognitive function, after appropriate weighting for differences between trials by duration of treatment and extent of homocysteine reduction. The analyses for global cognitive function showed no significant heterogeneity between the effects on cognitive aging in the 11 different trials (Figure 3), although some heterogeneity was seen in the findings for the cognitive-domain trials, which was mainly attributable to the FACIT trial (Figure 1).

The instruments used to assess cognitive function differed between trials, which could have contributed heterogeneity. Memory contributed approximately one-third of the weight in domain-composite scores and only approximately one-fifth of the weight in MMSE scores but approximately half of the weight in TICS-M scores (21). In addition, the domain-composite global cognitive score from FACIT included a higher proportion of tests of speed and evaluated more domains of speed (eg, sensorimotor, complex, and information processing speed) than in any other trial. Thus, the possibility that homocysteine lowering benefits solely these specific domains cannot be excluded. However, overall in the present meta-analysis, allocation to B vitamins had no significant effect on any of the individual domains of global cognitive function that could be assessed. Advances in functional neuroimaging may identify indicators of various aspects of cognitive aging and help provide more objective assessments of cognitive function (and calibration of instruments) for future studies.

The FACIT trial examined the effects of lowering homocysteine concentrations with a low dose of folic acid of 0.8 mg daily on 5 different cognitive domains and reported significant effects on the 3-y change in memory equivalent to cognitive performance of someone that is 4.7 y (95% CI: 1.1, 8.3 y) younger in age in 818 healthy participants with homocysteine concentrations ≥13 μmol/L (10). On average, participants in the FACIT trial were younger (60 compared with 66 y) and had lower mean plasma folate concentrations (12 compared with 15 nmol/L) than those in all of the global cognition trials. However, the present study found no effect of supplementation on MMSE-type scores in subgroups with lower folate concentrations or at younger ages (Supplemental Figure 3 under “Supplemental data” in the online issue). In addition, the participants in the FACIT trial were individuals with elevated homocysteine concentrations and normal serum vitamin B-12 concentrations (≥200 pmol/L), but the present meta-analysis found no heterogeneity in the effects of supplementation among those with vitamin B-12 concentrations <250 pmol/L compared with those with higher plasma vitamin B-12 concentrations (Supplemental Figure 4 under “Supplemental data” in the online issue). Although it is possible that the results of the FACIT trial may reflect some special attributes of the FACIT trial population or design (eg, correction of folate deficiency in individuals after excluding those with vitamin B-12 deficiency), the present meta-analysis does not provide any support for such an interpretation. Hence, in the absence of any consistent effects in different subgroups or over different cognitive domains, the differences between the results in FACIT and the other trials may be due to chance.

Consistent with previous reports (26, 27), the present study showed that, for the domain-composite score, change in *z* score offered some advantage over end-treatment *z* scores. The ratios of the variances of these 2 measures were 1:6 to 1:3, and hence (because power is a function of the variance of an individual observation divided by the number of observations) the use of change in domain-composite score equates to having 3–6 times as many individuals compared with the use of the end-treatment domain-composite score. However, for the MMSE-type scores, the variances of the change in score and end-treatment score were similar and so little would be gained by using change in MMSE-type *z* score.

Both domain-composite and MMSE-type scores showed strong inverse associations with age, but the relation was ∼50% steeper for the domain-composite score, suggesting that this was a somewhat more sensitive measure. However, offsetting these advantages of the change in domain-composite score were the longer durations and greater study sizes that were feasible in trials carrying out end-treatment MMSE-type assessments. An examination of the SEs of the estimates in Figure 3 indicates how the global cognitive measure, number of participants and duration of treatment influenced the relative contributions from individual trials. The FACIT trial (10), the largest of the cognitive-domain trials with 818 participants, contributed 2454 equivalent person-years, whereas SEARCH, the largest of the global cognition trials with 8891 participants (17), contributed 63,439 equivalent person-years. In FACIT, the SE of the effect of treatment was 0.034, whereas in SEARCH the SE was 0.021. Thus, the SE of SEARCH was approximately two-thirds that for FACIT, showing how a larger number of participants can offset a less stringent measure of global cognitive function. Moreover, for comparisons of the effect of treatment on global cognitive function per year at a 25% homocysteine reduction, the SE of the SEARCH result was only one-fifth of that for FACIT, showing the additional relevance of longer duration of treatment (Supplemental Figure 2 under “Supplemental data” in the online issue).

Trials in persons selected on the basis of a prior diagnosis of Alzheimer disease or cognitive impairment or depression (28–30) were excluded because the effects of treatment in people with established cognitive impairment may differ from those in the general population. One trial in 299 Australian participants (31) published after the specified cutoff for inclusion also reported no significant effects of B vitamins on cognitive function, but in a sensitivity analysis including the published results of this trial with all other trials, the results for the effects of B vitamins on cognitive aging were unaltered (Supplemental Table 6 under “Supplemental data” in the online issue).

The doses of folic acid (0.4–2.5 mg) used in the individual trials included in the present meta-analysis exceeded those required for maximal reduction in homocysteine concentrations. The possibility that high doses of folic acid might cause harm cannot be excluded, albeit there is no evidence of harm in previous trials (32). Except for one trial (10), all of the trials added vitamin B-12 (0.4–1 mg), which produces further homocysteine reduction and, with a daily dose of ∼1 mg in 4 trials (7, 12, 14, 17), should also correct for any undetected vitamin B-12 deficiency (assuming that it is reversible) (33). On the basis of findings of observational studies, it was anticipated that supplementation with B vitamins might slow the rate of cognitive aging (2–5). One-third of adults in the United States (34) and one-fifth of adults in the United Kingdom (35) report taking daily multivitamin supplements containing folic acid in the belief that they have beneficial effects for health, including prevention of cognitive aging. However, the claims that lowering homocysteine can prevent cognitive aging (10, 36, 37) within just a few years of treatment are not supported by the present meta-analysis. The null results are not influenced by selective survival of participants to have cognitive testing at the end of the study period, because supplementation with B vitamins had no effect on overall mortality (22).

Although trials used combinations of B vitamins (vitamin B-12 and vitamin B-6 in addition to folic acid) (22), it is unlikely that this would obscure any effects of folic acid alone on cognitive aging. Overall, homocysteine-lowering treatment with B vitamins did not substantially slow the rate of cognitive aging in older people with or without vascular disease, irrespective of how cognitive function was assessed. As with ischemic heart disease (22, 38), the discrepant results of the observational studies and the randomized trials for the effects of B vitamins on cognitive function suggest that elevated plasma homocysteine is probably a marker of underlying cognitive aging rather than a causal risk factor (39).

## Supplementary Material

Supplemental data

## Footnotes

6 Abbreviations used: FACIT, Folic Acid and Carotid Intima-Media Thickness; MMSE, Mini-Mental State Examination; SEARCH, Study of the Effectiveness of Additional Reductions in Cholesterol and Homocysteine; TIA, transient ischemic attack; TICS, Telephone Interview for Cognitive Status; TICS-M, Telephone Interview for Cognitive Status–Modified.

## APPENDIX A

### The following investigators were members of the B-Vitamin Treatment Trialists’ Collaboration

Secretariat: Robert Clarke, Derrick Bennett, Sarah Parish, Sarah Lewington, Jim Halsey, and Rory Collins (Clinical Trial Service Unit) and Alan D Dangour (London School of Hygiene and Tropical Medicine).

Cognitive-domain trials (ordered by number of participants): McMahon: Murray Skeaff, Jennifer McMahon, Tim Green, Jim Mann, Robert Knight, and Sheila Williams; Lewerin: Catharina Lewerin and Herman Nilsson Ehle; Eussen: Simone JPM Eussen, Lisette C de Groot, Wija A van Staveren, Willibrord Hoefnagels, and Liesbeth W Joosten; Stott: David J Stott.

Global cognition trials—SEARCH: Jane Armitage and Rory Collins; VITATOPS: Graeme J Hankey; WAFACS: JoAnn E Manson, William Christen, and Francine Grodstein; VISP: James Toole, M Rene Malinow, Lloyd Chambless, J David Spence, Luther Pettigrew, Virginia Howard, Elizabeth Sides, Chin-Hua Wang, and Meir Stampfer; SU.FOL.OM3: Pilar Galan and Serge Hercberg; HOPE-2: Eva Lonn and Salim Yusuf.
