# Supplementary material for: Effects of homocysteine lowering with B vitamins on cognitive aging: meta-analysis of 11 trials with cognitive data on 22,000 individuals1
Source: Am J Clin Nutr. 2014 Jun 25;100(2):657–66. doi: 10.3945/ajcn.113.076349 (PMC4095663; doi:10.3945/ajcn.113.076349)
Supplement: Supplemental data [file supp_100_2_657__index.html]

Effects of homocysteine lowering with B vitamins on cognitive aging: meta-analysis of 11 trials with cognitive data on 22,000 individuals — Supplemental data 

# Effects of homocysteine lowering with B vitamins on cognitive aging: meta-analysis of 11 trials with cognitive data on 22,000 individuals

## Supplemental data

**Files in this Data Supplement:**

- Supplemental data - Text, Tables 1-6 and Figures 1-5
