# Supplementary material for: Effects of homocysteine lowering with B vitamins on cognitive aging: meta-analysis of 11 trials with cognitive data on 22,000 individuals1
Source: Am J Clin Nutr. 2014 Jun 25;100(2):657–66. doi: 10.3945/ajcn.113.076349 (PMC4095663; doi:10.3945/ajcn.113.076349)
Supplement: Supplemental data [file 113.076349_ajcn076349SupplementaryData1.pdf]

Supplemental Material

Page 1: Contents list. (Click on an underlined item to jump to it)

P2: Supplemental Methods. Standard approach to combining individual domain tests and inclusion of published results from the FACIT trial.

P4: Supplemental Table 1. Details of different cognitive tests used in the cognitive-domain trials by domain as defined within the trial.

P6: Supplemental Table 2. Factors used to re-scale individual specific domains, domain-composite and MMSE-type global cognitive function scores.

P7: Supplemental Table 3. Daily doses of B-vitamins in individual trials.

P8: Supplemental Table 4. Median plasma levels of folate and homocysteine before and after study treatment.

P9: Supplemental Table 5. Median plasma levels of vitamin B12 before and after study treatment.

P10: Supplemental Table 6. Effects of B-vitamins on cognitive aging in all available trials (including Ford) after taking account of duration of treatment and percent homocysteine reduction.

P11: Supplemental Figure 1. Screening and selection of included trials.

P12: Supplemental Figure 2. Effects of B-vitamins on the memory and executive function domains of cognitive function assessed using change from baseline.

P13: Supplemental Figure 3. Effects of B-vitamins on the MMSE-type global cognitive function score in cognitive-domain trials assessed using change from baseline.

P14: Supplemental Figure 4. Effects of B-vitamins on MMSE-type global cognitive function score at the end of the treatment period in various subgroups in global cognition trials.

P15: Supplemental Figure 5. Effects of B-vitamins on cognitive aging using MMSE-type global cognitive function score in all available trials after taking account of duration of treatment and percent homocysteine reduction.

Supplemental Methods

Standard approach to combining individual domain tests

The Z-scores for each separate test were estimated by subtracting the mean and dividing by the standard deviation of the pooled results before and after treatment. The overall scores for the domains of memory, speed and executive were calculated from the average of the component Z-scores as defined within each individual trial. The domain-composite global cognitive function score was calculated from the average of the three domain-specific scores within each trial. [1, 2]

Inclusion of published results from the FACIT trial

The FACIT trial declined to contribute individual participant data, but it was possible to estimate most of the required statistics from the tables presented in their comprehensive original publication,[1] and hence the FACIT results are included in the present meta-analysis. Details of the methods used to include the published results of FACIT in the present meta-analysis are provided below:

i) Domain-specific and domain-composite global cognitive function: The end-treatment overall SD of the Z-score for each domain and domain-composite (referred to as "global cognitive function" in their paper) was computed from the sums of squares of the active and placebo group SDs at year 3 that were provided in Table 5 (p215) of the published report. This overall SD was used as the scaling factor. Reported score results (differences and SEs) were rescaled by dividing by the scaling factor as the equivalent of the rescaling applied to individual scores in the other studies.

ii) Homocysteine, Folate and Vitamin B12: For the FACIT trial the homocysteine (tHcy), folate and vitamin B12 data were abstracted from means, SDs and numbers reported in Table 3 of their published report (p214). The percent tHcy reduction was based on the difference between the percent reductions in the folate and placebo groups (i.e. $100*[(H_{3f} – H_{0f})/H_{of}$ $-(H_{3p} – H_{0p})/H_{0p}]$ where H_{0f} and H_{0p} are homocysteine levels at baseline in folic acid and placebo groups, respectively; H_{3f} and H_{3p} are homocysteine levels at year 3 in folic acid and placebo groups, respectively.

iii) Variance of global cognitive function scores in placebo allocated participants with repeat assessments: . The variance of change in domain-composite global cognitive function in the placebo group was re-scaled as in (i).

(iv) *Age association with domain-composite global cognitive function:* The association of age with domain-composite global cognitive function, was estimated from the reported result that the effect of 3 years of folic acid supplementation, given as 0.05 in Table5, corresponded to a cognitive performance of someone 1.5 years younger (p213). Thus, this yielded the change of FACIT score with age as -0.05/1.5=-0.033 per year and a change in our rescaled score of -0.033/0.6975=-0.048 per year..

All these manipulations are close summary equivalents to the procedures applied to individual participant data.

References

1. Durga J, van Boxtel MP, Schouten EG *et al*. Effect of 3-year folic acid supplementation on cognitive function in older adults in the FACIT trial: a randomised, double blind, controlled trial. Lancet 2007; 369(9557):208-16.

2. Lewerin C, Matousek M, Steen G, Johansson B, Steen B, Nilsson-Ehle H. Significant correlations of plasma homocysteine and serum methylmalonic acid with movement and cognitive performance in elderly subjects but no improvement from short-term vitamin therapy: a placebo-controlled randomized study. Am J Clin Nutr 2005; 81(5):1155-62.

Supplemental Table 1: Details of different cognitive tests used in each cognitive-domain trial by domain as defined within the study

Trial	Name of cognitive test	Cognitive domain as defined in study	Brief description of the test
Eussen	Finger Tapping	Speed	Computerised task, press a single button as often as possible within 30 seconds.
Eussen	Motor Planning_2	Speed	Computerised task, press a lit button out of 3 buttons as often as possible within 30 seconds.
Eussen	Motor Planning_3	Executive function	Computerised task, press a button immediately adjacent to a lit button
Eussen	Figure of Rey – copy	Praxis	Copy the complex figure of Rey from an example
Eussen	Figure of Rey - immediate recall	Memory	Draw the complex figure of Rey without the example immediately after the copy
Eussen	15 Word Learning –immediate recall	Memory	15 words are read 5 times and in between the participant recalls the words
Eussen	Retian Trail Making Test Part A	Speed	Randomly placed numbers have to be connected with a line as fast as possible
Eussen	Reitan Trail Making Test Part B	Executive function	Randomly numbers and letters have to be connected with a line as fast as possible
Eussen/Lewerin	Digit Span Forward	Attention	Repeat a string of digits in original order
Eussen/Lewerin	Digit Span Backward	Memory	Repeat a string of digits in reverse order
Eussen	Raven Progressive Matrices	Executive function	Chose a design that fits into a matrix for 24 matrices
Eussen/FACIT	Stroop 1	Executive function	Read names of colours red, green yellow, blue
Eussen/FACIT	Stroop 2	Executive function	Name coloured bocks red green, yellow, blue
Eussen/FACIT	Stroop 3	Executive function	Name colour of the ink rather than the word
Eussen	Figure of Rey - delayed recall	Memory	Draw the complex figure of Rey without the example 30 minutes after the copy
Eussen	15 Word Learning - delayed recall	Memory	Recalls the words of the 15 word learning test the participants remembered
Eussen	15 Word Learning	Memory	30 words are read of which the participant has to recognise the original 15 words
Eussen	Similarities Weschler Adult Intelligence Scale	Executive function	Mention similarities between 5 pairs of nouns
Eussen	Verbal Fluency, letter	Executive function	List as many nouns beginning with letter P or G as possible in 2 minutes
Eussen/FACIT	Verbal Fluency, animal	Executive function	List as many animals as possible in 2 minutes.
Lewerin	Identical forms	Speed	Contains 60 items of identification. For each item, a complex figure is compared with 5 other figures, and the one that is identical is marked
Lewerin	Visual reproduction	Memory	4 drawings are shown to the tested subject to be remembered and reproduced
Lewerin	Synonyms	Executive function	The subjects have to select from among 5 words a synonym for a given word
Lewerin	Block design	Executive function	The test consists of 7 designs that have to be made out of red, white, and red and white blocks. Bonuses are given for rapid performance.
Lewerin	Digit symbol	Speed	The subjects are asked to replace digits with symbols according to an existing code with a time limit of 90 seconds.
Lewerin	Thurstones's Picture Memory Test	Memory	Subjects look at 28 pictures consecutively, which are presented at a rate of every 5 seconds; they are later asked to identify the picture among 4 similar pictures.
Lewerin	Figure classification	Executive function	In each item 5 figures are given the figure that is different from the others should be marked by the subject.
McMahon	Rey Auditory Verbal Learning Test Trials I - V	Memory	Consists of five presentations of a 15-word list. Participants are asked to recall as many words as possible on the lists after each presentation.
McMahon	Rey Auditory Verbal Learning Test Delayed Trial VII	Memory	Delayed recall may be examined with a seventh recall trial after a 20-30 minute delay.

Supplemental Table 1: Details of different cognitive tests used in each cognitive-domain trial by domain as defined within the study

Trial	Name of cognitive test	Cognitive domain as defined in study	Brief description of the test
McMahon	Weschler Paragraph Recall tests	Memory	Asked to recall the main details after hearing a short story (paragraph) either immediately or after about an hour without warning.
McMahon	Controlled Oral Word Association Test of the Multilingual Aphasia Examination	Executive function	Requires participants to generate as many words as possible with a specified initial letter in 60 seconds
McMahon	Category Word Fluency Test	Executive function	Total number of words generated in a specific category in 60 seconds. For example subjects asked to generate words from three specified categories (animals, fruit and vegetables and means of transportation)
McMahon	Reitan Trail Making Test Part B	Speed	Randomly numbers and letters have to be connected with a line as fast as possible
FACIT	Word Learning Test	Memory	Participants asked to memorise 15 commonly used monosyllabic words presented in a fixed sequence of 2 seconds. Immediately after the 15 words are presented the subjects are asked to recall the words.
FACIT	Concept shifting tests	Executive function	A timed tests with 4 subtests that measure switching between two psychological concepts
FACIT	Letter Digit Substitution Test	Speed	Nine different letters are assigned a unique number (1-9) the participants are presented with a random series of letters and asked to add the corresponding digit to the letters in 90 seconds.

Supplemental Table 2: Factors used to re-scale individual specific domains, domain-composite and MMSE-type global cognitive function scores

	End-treatment SD values used to re-scale domain-specific and global cognitive scores				
**Trial (Reference)**	**Domain-composite global cognitive scores**	**Memory**	**Executive function**	**Speed**	**MMSE-type global cognitive scores**
Cognitive-domain trials					
Eussen (7)	0.392	0.653	0.502	0.736	0.950
Lewerin (8)	0.680	0.704	0.777	0.865	
McMahon (9)	0.605	0.736	0.712	0.899	0.960
FACIT (10)[1]	0.698	0.963	0.991	0.880	
Global cognition trials					
Stott (11)					0.985
HOPE-2 (12)					0.982
SU.FOL.OM3 (13)					0.953
WAFACS (14)					0.976
VISP (15)					0.983
VITATOPS (16)					0.983
SEARCH (17)					0.949

[1] Published data only.

Supplemental Table 3: Daily doses of B-Vitamins in individual trials

Trial (Reference)	Number with cognitive function measured at baseline	Number (%) with cognitive function at end treatment	Inclusion criteria: Age range (years)	Inclusion criteria: B12 (pmol/L)	Prior disease[1]	Country	Daily dose of B-vitamins (mg): Folic acid	Daily dose of B-vitamins (mg): Vitamin B12	Daily dose of B-vitamins (mg): Vitamin B6
Cognitive-domain trials									
Eussen (7)[2]	130	108 (83)	70+	100 -300	Nil	Netherlands	0.4	0.99	-
Lewerin (8)	202	183 (91)	N/A	N/A	Nil	Sweden	0.8	0.50	3
McMahon (9)	273	248 (91)	65+	N/A	Nil	New Zealand	1.0	0.50	10
FACIT (10)[3]	818	801 (98)	50 -70	200+	Nil	Netherlands	0.8	-	-
**Sub-total**	**1423**	**1340 (94)**							
Global cognition trials									
Stott (11)[2]	185	167 (90)	65+	185+	Stroke[4]	UK	2.5	0.40	25
HOPE-2 (12)	1327	1245 (94)	55+	N/A	CVD	Canada/Europe	2.5	1.00	50
SU.FOL.OM3 (13)[2]	-	1309 (-)	45 -80	N/A	CHD	France	0.6	0.02	3
WAFACS (14)	2007	1756 (87)	65+	N/A	CVD	USA	2.5	1.00	50
VISP (15)	3680	2653 (72)	35+	N/A	Stroke	USA/UK [5]	2.5	0.40	25
VITATOPS (16)	-	4410 (-)	45 -80	N/A	Stroke	20 countries [6]	2.0	0.50	25
SEARCH (17)	-	8891 (-)	18 -80	N/A	CHD	UK	2.0	1.00	-
**Sub-total**	**7199**	**20431 (-)**							

[1] CVD: Prior cardiovascular disease or increased risk of cardiovascular disease; CHD: Coronary Heart Disease.
[2] Refers to group allocated B-vitamins vs placebo.
[3] Published data only.
[4] About 60% of participants had a prior history of stroke.
[5] 3634 from United States, 46 from United Kingdom.
[6] Australia, Austria, Belgium, Brazil, Hong Kong, India, Italy, Malaysia, Moldova, Netherlands, New Zealand, Pakistan, Philippines, Portugal, Republic of Georgia, Serbia & Monte Negro, Singapore, Sri Lanka, UK, USA.

Supplemental Table 4: Median plasma levels of folate and homocysteine before and after study treatment

Trial (Reference)	Number with cognitive function measured at baseline	Number (%) with cognitive function at end treatment	Folate (nmol/L)				Homocysteine (μmol/L)			
			B-vitamins		Placebo		B-vitamins		Placebo	
			Before (N)	After(N)	Before(N)	After(N)	Before(N)	After(N)	Before(N)	After(N)
Cognitive-domain trials										
Eussen (7)[1, 2]	130	108 (83)	-	-	-	-	14.4 (63)	9.0 (51)	15.0 (63)	14.0 (57)
Lewerin (8)	202	183 (91)	14 (139)	61 (126)	16 (70)	16 (67)	16.7 (139)	11.3 (126)	15.7 (70)	15.8 (67)
McMahon (9)	273	248 (91)	18 (127)	75 (124)	20 (125)	19 (121)	15.8 (127)	8.7 (127)	16.0 (126)	13.0 (126)
FACIT (10)[3]	818	801 (98)	12 (405)	76 (394)	12 (413)	13 (406)	13.0 (405)	10.1 (393)	12.9 (412)	13.4 (406)
**Sub-total**	**1423**	**1340 (94)**	**13 (671)**	**67 (644)**	**13 (608)**	**14 (594)**	**14.0 (734)**	**10.0 (697)**	**13.7 (671)**	**13.5 (656)**
Global cognition trials										
Stott (11)[2]	185	167 (90)	-	-	-	-	14.8 (92)	10.7 (89)	15.1 (93)	15.7 (86)
HOPE-2 (12)	1327	1245 (94)	28 (287)	45 (288)	29 (290)	23 (289)	10.7 (568)	9.0 (292)	10.7 (559)	11.9 (294)
SU.FOL.OM3 (13)[2]	-	1309 (-)	15 (1204)	36 (573)	16 (606)	15 (272)	13.0 (1228)	11.6 (571)	12.6 (614)	14.5 (276)
WAFACS (14)	2007	1756 (87)	20 (51)	93 (51)	21 (55)	39 (55)	12.9 (51)	11.1 (51)	13.6 (55)	13.4 (55)
VISP (15)	3680	2653 (72)	23 (1681)	63 (1407)	23 (1704)	23 (1437)	12.3 (1800)	9.5 (1505)	12.3 (1822)	11.6 (1546)
VITATOPS (16)	-	4410 (-)	21 (118)	32 (84)	19 (113)	20 (81)	12.5 (604)	9.6 (580)	12.8 (601)	13.0 (584)
SEARCH (17)	-	8891 (-)	14 (4286)	52 (686)	14 (4331)	14 (746)	12.3 (4365)	9.6 (4150)	12.3 (4414)	13.4 (4247)
**Sub-total**	**7199**	**20431 (-)**	**15 (7627)**	**46 (3089)**	**16 (7099)**	**19 (2880)**	**12.3 (8708)**	**9.7 (7238)**	**12.2 (8158)**	**13.0 (7088)**

[1] Median red cell folate levels before and after treatment were 528 and 1413 nmol/L, respectively, in those allocated B-vitamins and 566 and 618 nmol/L respectively, in those allocated control.
[2] Refers to group allocated B-vitamins vs placebo.
[3] Published data only.
Folate was measured by immunoassay in all trials except for Lewerin and VISP (which used a radioassay) and McMahon (which used a microbiological assay).

Supplemental Table 5: Median plasma levels of vitamin B12 before and after study treatment

Trial (Reference)	No. with cognitive function measured at baseline	No. (%)with cognitive function at end treatment	Vitamin B12 (pmol/L)			
			B-vitamins		Placebo	
			Before(N)	After(N)	Before(N)	After(N)
Cognitive-domain trials						
Eussen (7)[1]	130	108 (83)	196 (62)	583 (51)	173 (63)	175 (55)
Lewerin (8)	202	183 (91)	290 (139)	532 (125)	330 (70)	321 (67)
McMahon (9)	273	248 (91)	267 (127)	543 (124)	265 (126)	242 (124)
FACIT (10)[2]	818	801 (98)	290 (405)	264 (394)	286 (413)	273 (406)
**Sub-total**	**1423**	**1340(94)**	**277 (733)**	**362 (694)**	**279 (672)**	**261 (652)**
Global cognition trials						
Stott (11)[1]	185	167 (90)	336 (92)	641 (88)	343 (93)	365 (81)
HOPE-2 (12)	1327	1245 (94)	277 (284)	885 (281)	263 (286)	248 (278)
SU.FOL.OM3 (13)[1]		1309(-)	359 (1227)	499 (575)	376 (612)	370 (274)
WAFACS (14)	2007	1756 (87)	333 (22)	-	314 (30)	-
VISP (15)	3680	2653 (72)	320 (1681)	541 (1424)	324 (1707)	362 (1437)
VITATOPS (16)	-	4410 (-)	283 (243)	327 (214)	289 (251)	161 (209)
SEARCH (17)	-	8891 (-)	260 (4287)	739 (686)	263 (4331)	261 (746)
**Sub-total**	**7199**	**20431(-)**	**289 (7836)**	**582 (3268)**	**287 (7310)**	**318 (3025)**

[1] Refers to group allocated B-vitamins vs placebo.
[2] Published data only.

Supplemental Table 6: Effects of B-vitamins on cognitive aging in all available trials (including Ford) after taking account of duration of treatment and percent homocysteine reduction

	Equivalent study years at 25% tHcy reduction		Global cognitive function Z-score difference (SE) by allocated treatment arm (B-vitamin minus placebo)		
Trial (Reference)	Study years	Person years	Overall in study	Per year at 25% tHcy reduction	Years of cognitive aging equivalent (95% CI)
**Cognitive-domain trials, assessing change in domain-composite score**					
Eussen (7)	0.64	29	-0.167 (0.139)	-0.2606 (0.2179)	4.83 (-3.08, 12.73)
Lewerin (8)	0.39	55	-0.101 (0.085)	-0.2561 (0.2170)	4.74 (-3.14, 12.62)
McMahon (9)	2.84	553	-0.170 (0.069)	-0.0599 (0.0245)	1.11 (0.22, 2.00)
Ford (31)	2.66	598	0.055 (0.127)	0.0206 (0.0478)	-
FACIT (10)	3.13	2454	0.071 (0.034)	0.0228 (0.0108)	-0.42 (-0.81, -0.03)
Subtotal: cognitive (excluding Ford)	-	-	0.003 (0.028)	0.0082 (0.0098)	-0.15 (-0.51, 0.20)
**Global cognition trials, assessing end of treatment MMSE-type score**					
Stott (11)	1.13	148	0.009 (0.155)	0.0082 (0.1365)	-0.23 (-7.66, 7.20)
Ford (31)	2.66	598	-0.105 (0.129)	-0.0396 (0.0485)	1.10 (-1.54, 3.74)
HOPE-2 (12)	4.64	6090	-0.002 (0.057)	-0.0004 (0.0122)	0.01 (-0.65, 0.68)
WAFACS (14)	2.95	10379	0.006 (0.048)	0.0019 (0.0162)	-0.05 (-0.93, 0.83)
SU.FOL.OM3 (13)	3.88	6152	-0.049 (0.059)	-0.0128 (0.0153)	0.35 (-0.48, 1.18)
VISP (15)	1.30	5193	0.013 (0.039)	0.0101 (0.0299)	-0.28 (-1.91, 1.35)
VITATOPS (16)	3.32	14256	0.001 (0.030)	0.0004 (0.0091)	-0.01 (-0.51, 0.48)
SEARCH (17)	8.45	63439	-0.011 (0.021)	-0.0013 (0.0025)	0.04 (-0.10, 0.17)
Subtotal: other	-	-	-0.006 (0.014)	-0.0014 (0.0023)	0.04 (-0.09, 0.16)
**All**	-	-	-0.004 (0.012)	-0.0009 (0.0023)	0.02 (-0.10, 0.14)

The study by Ford was a double-blind randomized controlled trial of 299 men randomized to receive either B-vitamins or placebo for 2 years. This study reported both change from baseline results and end-treatment results for global cognitive function as measured by the MMSE and ADAS-cog. The numbers in this table are based on the MMSE as this was used in other studies. The percent homocysteine reduction observed was 33.2% for those receiving B-vitamins compared to those receiving placebo.

The overall global cognitive function Z-score difference (SE) using the ADAS-cog for the Ford study from the change from baseline analysis was 0.0272 (0.1289) with a per year at 25% tHcy reduction of 0.01023 (0.0485).

The years of cognitive aging equivalent (95% CI) for the Ford study using the end-treatment ADAS-cog was 1.52 (-1.12, 4.16) this gave a years of cognitive aging for all studies combined (95% CI) of -0.02 (-0.10, 0.14).

Supplemental Figure 1: Screening and selection of included trials

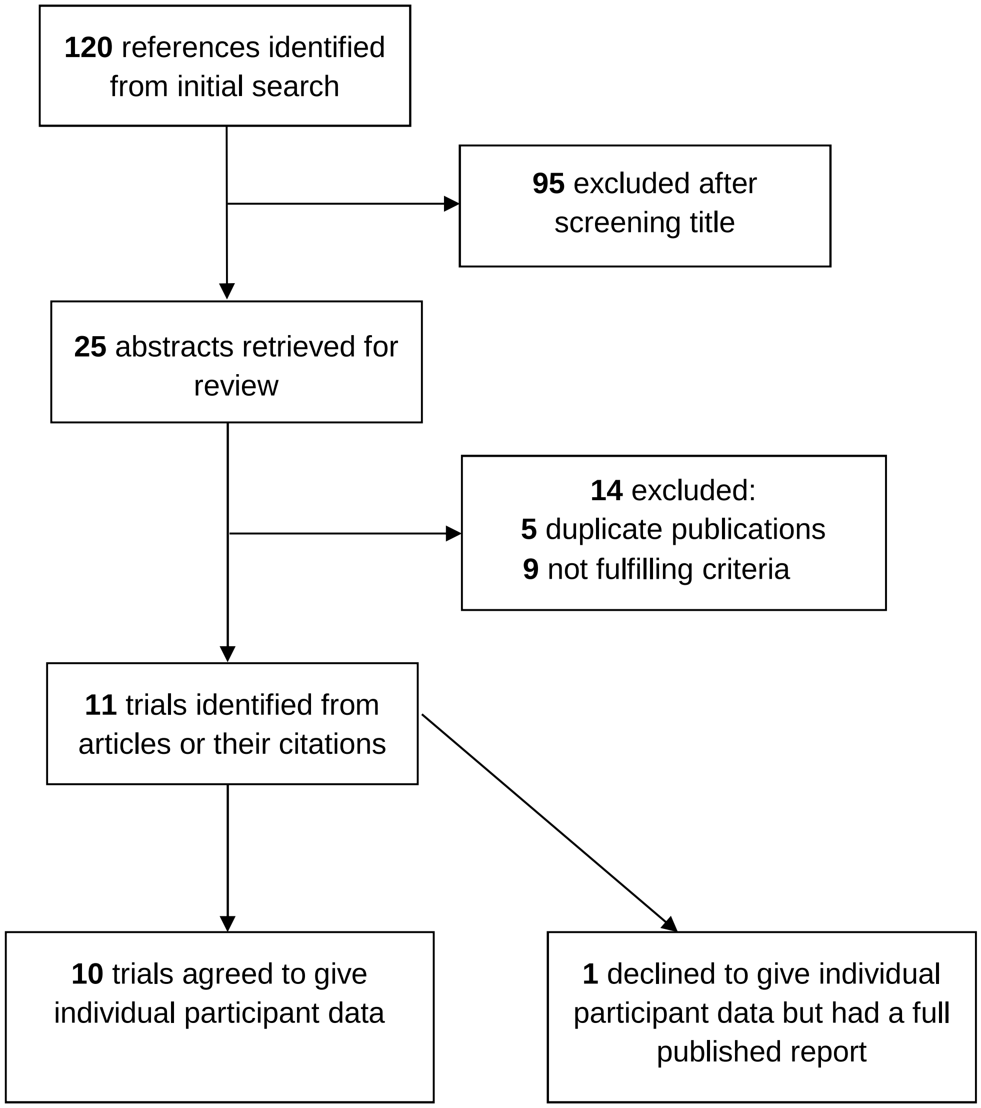

Supplemental Figure 2: Effects of B-vitamins on the memory and executive function domains of cognitive function assessed using change from baseline[1]

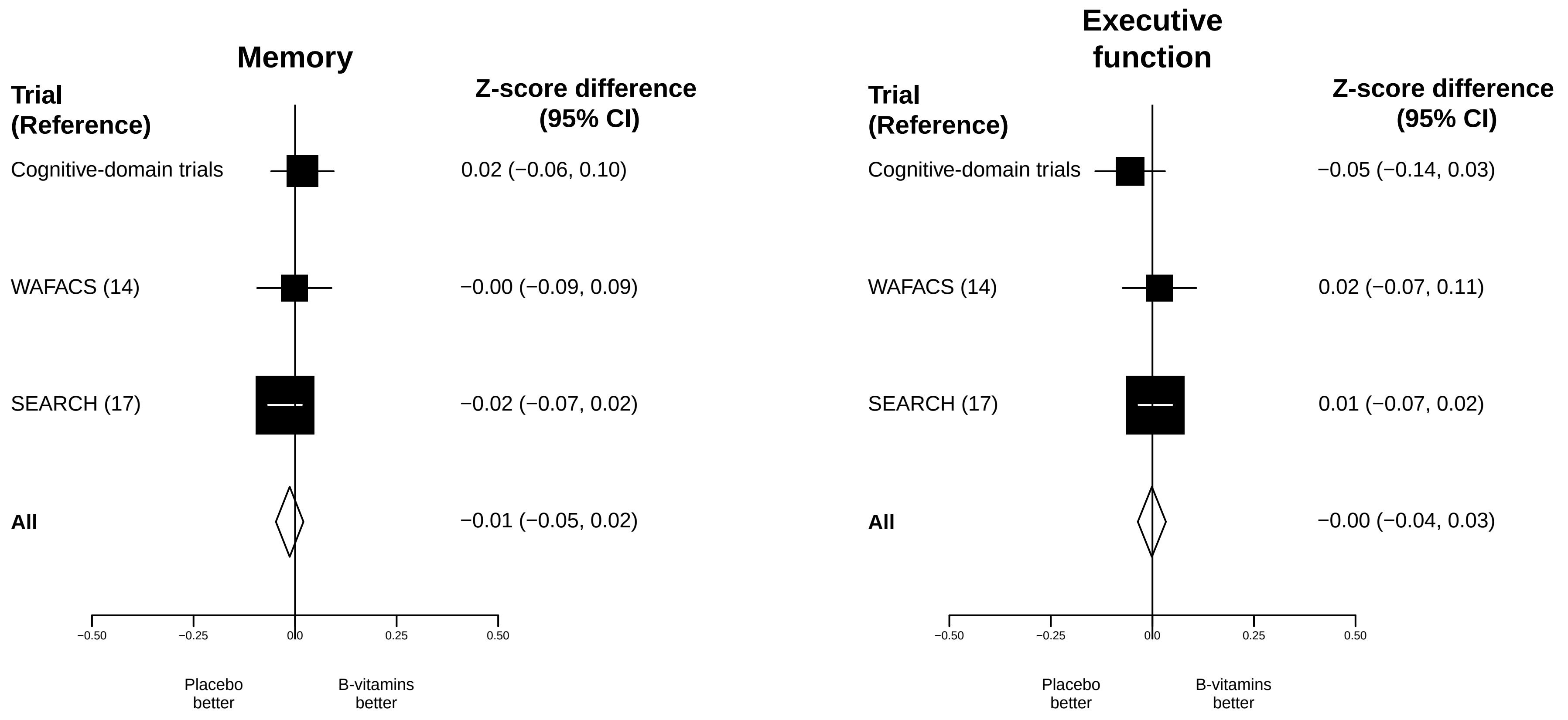

[1] All studies assessed by change from baseline except SEARCH, which was assesed at end of scheduled treatment

Supplemental Figure 3: Effects of B-vitamins on MMSE-type global cognitive function score in the cognitive-domain trials assessed using change from baseline

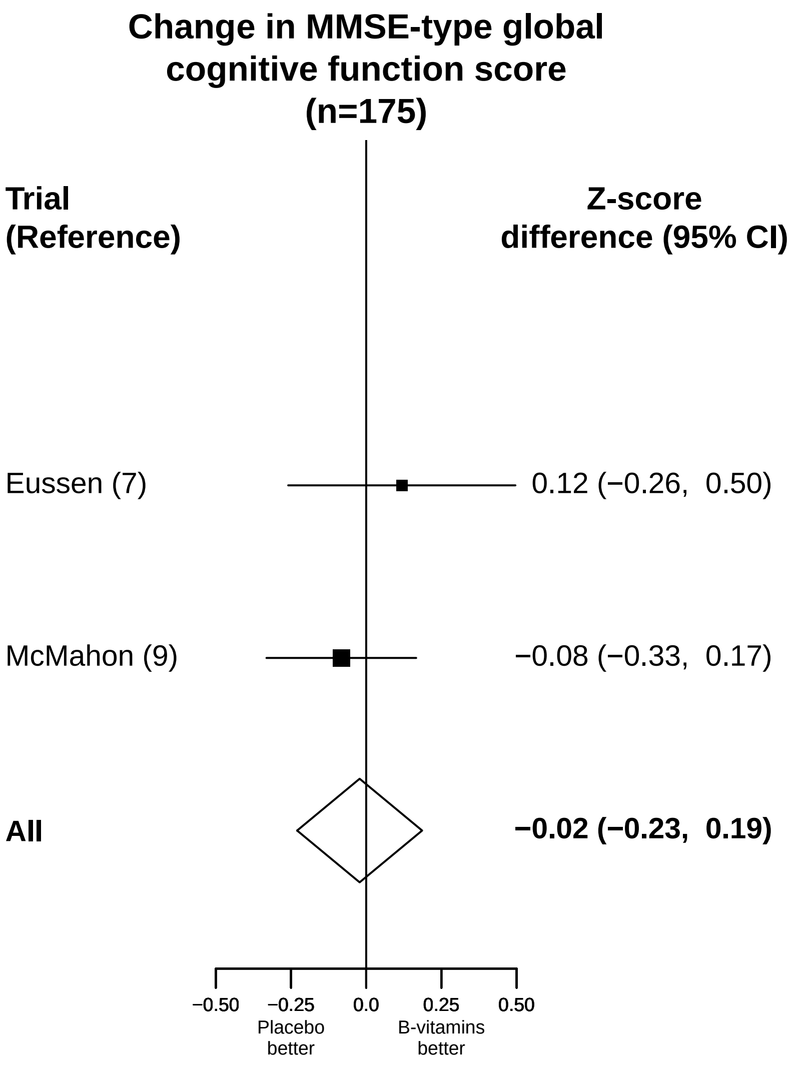

Supplemental Figure 4: Effects of B-vitamins on MMSE-type global cognitive function score at the end of the treatment period in various subgroups in global cognition trials

Subgroup	B-vitamins Z-score (N)	Placebo Z-score (N)	Z-score difference for global cognitive score (SE)
**Age at randomisation**			
<60	0.01 (3129)	0.01 (2924)	0.00 (0.03)
60−69	0.03 (3661)	0.03 (3475)	0.01 (0.02)
70+	−0.05 (3671)	−0.02 (3571)	−0.02 (0.02)
**Sex**			
Male	0.02 (7155)	0.01 (6843)	0.01 (0.02)
Female	−0.04 (3305)	−0.01 (3127)	−0.03 (0.02)
**Smoking status**			
Never	0.02 (2064)	−0.01 (2127)	0.03 (0.03)
Ex	−0.01 (4457)	0.01 (4361)	−0.02 (0.02)
Current	−0.10 (1621)	−0.06 (1223)	−0.04 (0.04)
Unknown	0.00 (2319)	−0.00 (2259)	0.00 (0.03)
**History of stroke**			
No	0.22 (6455)	0.24 (6147)	−0.02 (0.02)
Yes	0.01 (4006)	−0.01 (3823)	0.02 (0.02)
**Fortification**			
No	−0.19 (5410)	−0.18 (4987)	−0.01 (0.02)
Yes	0.00 (5051)	−0.00 (4983)	0.00 (0.02)
**Duration (years)**			
<3	−0.13 (2602)	−0.13 (2608)	0.01 (0.03)
3+	0.14 (7811)	0.16 (7313)	−0.01 (0.02)
Missing	0.13 (48)	−0.01 (49)	0.13 (0.20)
**Folate (nmol/L)**			
<10.0	0.01 (1933)	0.01 (1516)	−0.00 (0.03)
10.0−18.0	0.06 (2174)	0.09 (2213)	−0.02 (0.03)
18.0+	0.12 (2689)	0.11 (2664)	0.01 (0.03)
Missing	−0.01 (3665)	−0.00 (3577)	−0.01 (0.02)
**Vitamin B12 (pmol/L)**			
<250	0.06 (2597)	0.08 (2506)	−0.02 (0.03)
250−349	0.12 (2415)	0.09 (2252)	0.03 (0.03)
350+	0.12 (1960)	0.12 (1812)	0.00 (0.03)
Missing	−0.02 (3489)	−0.00 (3400)	−0.02 (0.02)
**Homocysteine (μmol/L)**			
<11.0	0.11 (2542)	0.13 (2419)	−0.02 (0.03)
11.0−14.0	0.06 (2796)	0.09 (2684)	−0.02 (0.03)
15.0+	0.03 (2351)	0.01 (2153)	0.02 (0.03)
Missing	−0.02 (2772)	−0.02 (2714)	0.00 (0.03)
**Baseline cognitive impairment** [1]			
Yes	−0.75 (293)	−0.81 (312)	0.05 (0.08)
No	0.39 (2133)	0.38 (2145)	0.01 (0.03)
Missing	−0.03 (8035)	−0.02 (7513)	−0.01 (0.02)
**All**	−0.00 (10461)	0.00 (9970)	−0.01 (0.01)

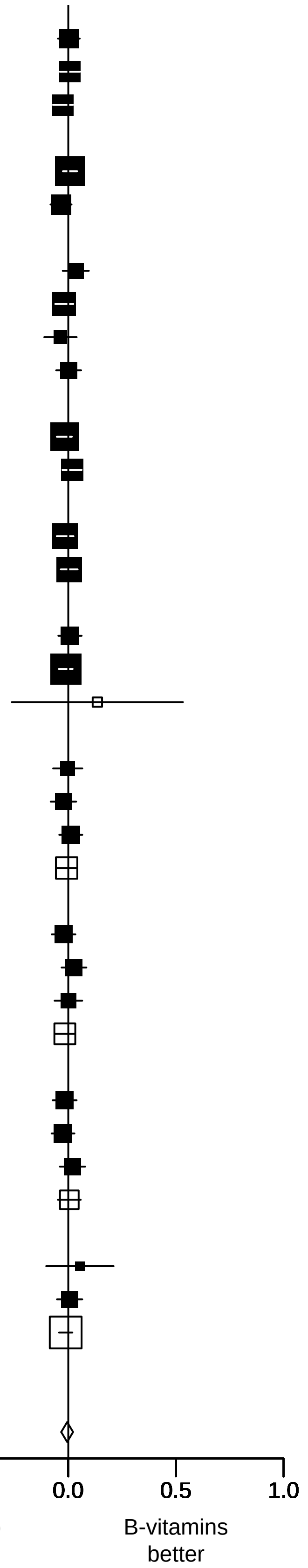

[1]Cognitive impairment defined as MMSE score of < 24, a TICS score of < 31, or a TICS-M score of < 22

Supplemental Figure 5: Effects of B-vitamins on cognitive aging using MMSE-type global cognitive function score in all available trials after taking account of duration of treatment and percent homocysteine reduction

Trial (Reference)	Years of cognitive aging equivalent (95% CI) [1]
Cognitive-domain trials	
Eussen (7)	−7.80 (−24.4, 8.86)
McMahon (9)	0.08 (−2.35, 2.51)
**Subtotal: cognitive**	**−0.09 (−2.49, 2.32)**
Global cognition trials	
Stott (11)	−0.23 (−7.66, 7.20)
HOPE−2 (12)	0.01 (−0.65, 0.68)
SU.FOL.OM3 (13)	0.35 (−0.48, 1.18)
WAFACS (14)	−0.05 (−0.93, 0.83)
VISP (15)	−0.28 (−1.91, 1.35)
VITATOPS (16)	−0.01 (−0.51, 0.48)
SEARCH (17)	0.04 (−0.10, 0.17)
**Subtotal: other**	**0.04 (−0.09, 0.16)**
**All**	**0.04 (−0.09, 0.16)**

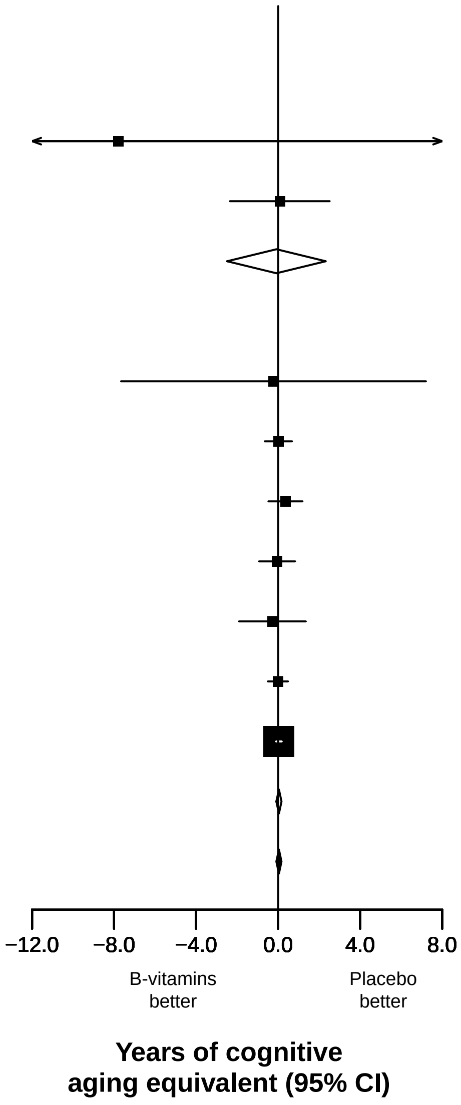

[1] The age association was based on 0.036 per year reduction in MMSE-type cognitive Z-score
